# Comparative transcriptomic analysis revealed adaptation mechanism of *Phrynocephalus erythrurus*, the highest altitude Lizard living in the Qinghai-Tibet Plateau

**DOI:** 10.1186/s12862-015-0371-8

**Published:** 2015-06-02

**Authors:** Yongzhi Yang, Lizhong Wang, Jin Han, Xiaolong Tang, Ming Ma, Kun Wang, Xiao Zhang, Qian Ren, Qiang Chen, Qiang Qiu

**Affiliations:** State Key Laboratory of Grassland Agro-ecosystem, School of Life Sciences, Lanzhou University, Lanzhou, Gansu China; Institute of Biochemistry and Molecular Biology, School of Life Sciences, Lanzhou University, Lanzhou, Gansu China

**Keywords:** Lizard, High altitude adaptation, Positive selection, Accelerate evolutionary rate, Hypoxia, UV damage

## Abstract

**Background:**

Organisms living at high altitudes must overcome three major environmental challenges: hypoxia, cold, and intense UV radiation. The molecular mechanisms that enable these challenges to be overcome have mainly been studied in endothermic organisms; relatively little attention has been paid to poikilothermic species. Here, we present deep transcriptome sequencing in two closely related lizards, the high altitude-dwelling *Phrynocephalus erythrurus* and the lowland-dwelling *P. putjatia*, to identify candidate genes under positive selection and to explore the convergent evolutionary adaptation of poikilothermic animals to high altitude life.

**Results:**

More than 70 million sequence reads were generated for each species via Illumina sequencing. *De novo* assembly produced 56,845 and 63,140 transcripts for *P. erythrurus* and *P. putjatia*, respectively. *P. erythrurus* had higher Ka/Ks ratios than *P. putjatia*, implying an accelerated evolutionary rate in the high altitude lizard lineage. 206 gene ontology (GO) categories with accelerated evolutionary rates and 43 candidate positively selected genes were detected along the *P. erythrurus* lineage. Some of these GO categories have functions associated with responses to hypoxia, energy metabolism and responses to UV damage. We also found that the high-altitude ranid frog *R. kukunoris* had higher Ka/Ks ratios than the closely related low-altitude frog *R. chensinensis*, and that the functional categories with accelerated evolutionary rates in *R. kukunoris* overlapped extensively with those detected along the *P. erythrurus* lineage.

**Conclusions:**

The mechanisms of high altitude adaptation in *P. erythrurus* were tentatively inferred. By comparing two pairs of low- and high-altitude poikilothermic species, we found that similar functional categories had undergone positive selection in high altitude-dwelling *Phrynocephalus* and *Rana* lineages, indicating that similar mechanisms of adaptation to high altitude might have evolved in both genera. Our findings provide important guidance for future functional studies on high altitude adaptation in poikilothermic animals.

**Electronic supplementary material:**

The online version of this article (doi:10.1186/s12862-015-0371-8) contains supplementary material, which is available to authorized users.

## Background

Survival at high altitudes is very challenging. Nevertheless, many native peoples and animals can thrive under the cold and hypoxic conditions associated with high altitude environments [1]. The molecular responses to high-altitude stress have been studied for over a century, but most of the previous studies focused on a single or a few candidate genes in model systems, which has limited our understanding of the molecular basis of adaptation in non-model systems [2–4]. The recent development of next-generation sequencing (NGS) technologies has significantly accelerated the speed of gene discovery and genomics studies, greatly facilitating evolutionary and ecological research on non-model organisms [5, 6]. Numerous studies on the adaptation of endothermic animals to high altitude have been published; species investigated at the genome scale include the yak (descendants of wild yak) [7], Tibetan wild boar [8], ground tit [9], snow leopard [10] and Tibetan mastiff [11], while transcriptome-scale studies have examined the blind subterranean mole rat [12], deer mice [13, 14] and rufous-collared sparrows [15]. These studies resulted in the identification of many genes associated with hypoxia tolerance that have undergone natural selection in high altitude-dwelling species or have different expression patterns in high altitude species between closely related low altitude dwellers. However, there have been few investigations into the mechanisms of high altitude adaptation in poikilothermic animals.

Toad-headed sand lizards of the genus *Phrynocephalus* are widespread in the Qinghai-Tibet Plateau (QTP) [16]. They comprise over 40 species and represent an important component of low and high-altitude desert ecosystems [17]. Despite increasing awareness of their ecological importance, the ecological niches and roles of *Phrynocephalus* species in lizard communities of extreme environments are poorly studied. *Phrynocephalus erythrurus*, which typically lives at altitudes of 4,500 m above sea level (a.s.l) or more, is considered to be the most high altitude-adapted lizard in the world [18]. It has a congener, *P. putjatia*, which is widely distributed across the Gobi desert and the semi-desert areas of the Qinghai Lake Basin, and commonly lives at lower altitudes of around 2,500 m.a.s.l [19]. The adaptations of these two *Phrynocephalus* species to their respective habitats are likely to have contributed significantly to species diversification within the genus, making *Phrynocephalus* an ideal system for studying high altitude adaptation in poikilothermic animals. Previous investigations have examined differences in the efficiency of ATP production in the mitochondria of sand lizard species dwelling at different altitudes, as well as differences in their metabolic activity and physiology [20, 21]. However, little is known about the molecular mechanisms that underpin adaptation to life at very high altitudes in this genus. Such studies have been hindered by the absence of genomic and transcriptomic resources for these non-model organisms.

Here we present a large scale, multi-tissue, multi-individual transcriptome for *P. erythrurus* and *P. putjatia* that was derived using RNA-seq. This transcriptome was created for two main reasons. First, to develop a molecular resource to support studies on sand lizards and make it available to the scientific community. Second, to identify adaptive evolutionary patterns in sand lizards, which will hopefully provide important insights into the genetic basis of high altitude adaptation and speciation. An additional goal of this study was to analyse transcriptomic data for ranid species dwelling at high and low altitudes [22], and to determine whether the evolutionary adaptations to life at high altitude in these cold-blooded organisms were functionally convergent with those seen in the sand lizards.

## Results and Discussion

### Sequencing and *De novo* assembly

**Fig. 1:**
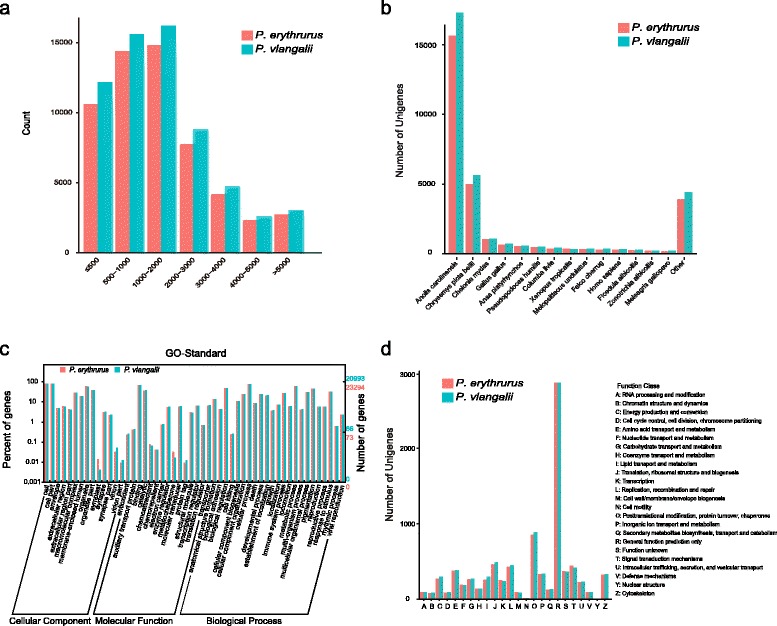
Overview of *P. erythrurus* and *P. putjatia* assemblies and annotations. **(a)** Length distribution of assembled transcripts in base pairs. **(b)** Top-Hit species distribution of BLASTx matches to unigenes. **(c)** Gene Ontology (GO) distributions of unigenes. **(d)** COG function classification of unigenes

A total of 80,538,854 and 79,852,538 sequence reads were generated for *P. erythrurus* and *P. putjatia*, respectively. After trimming adapter sequences and removing low-quality sequences, we obtained 71,958,336 and 70,420,332 clean reads, respectively. All of the clean reads were assembled into transcripts using the Trinity program and CD-HIT was used to cluster transcripts to obtain the final assembly. For *P. erythrurus*, 56,845 unigenes were obtained with an N50 size of 2,681 base pairs (bp) and a mean length of 1,735 bp. For *P. putjatia*, 63,140 unigenes were obtained with an N50 size of 2,691 bp and a mean length of 1,730 bp. Partial and complete open reading frames (ORFs) were predicted using the transdecoder script from the Trinity package, with a minimum length of 100 amino acids. Finally, 31,031 proteins were translated from the *P. erythrurus* assembly and 32,865 proteins from the *P. putjatia* assembly. These results are summarized in Table 1, and the length distribution of all transcripts is shown in Fig. 1a.

**Table 1:** Summary of transcriptome data for P. erythrurus and P. putjatia

	*P. erythrurus*	*P. putjatia*
Total number of reads	71,958,336	70,420,332
Read length (bp)	100	100
**Final Assembly**		
Total length of the final assembly (Mb)	98.62	109.26
Total number of transcripts	56,845	63,140
N50 length of assembly (bp)	2,681	2,691
Mean length of assembly(bp)	1,735	1,730
Median length of assembly(bp)	1,180	1,180
**Predicted Proteins**		
Total number of predicted proteins	31,031	32,865
N50 length of predicted proteins (AA)	569	553
Mean length of predicted proteins (AA)	397	384
bp = base pair; Mb = mega base pairs.

### Annotation

The complete set of unigenes from both species was queried against the NR, GO, Swiss-prot, COG and KEGG databases. In total, 29,495 (51.89 %) sequences from *P. erythrurus* and 32,823 (51.98 %) from *P. putjatia* yielded at least one significant match to an existing gene model in BLASTX searches (Table 2). Most of the unigenes that generated hits in the BLAST searches had relatively low E-values and high levels of similarity: 80.14 % of the *P. erythrurus* unigenes and 79.81 % of those from *P. putjatia* had E-values below 1E-50; while 83.25 % and 83.57 % of these two species’ unigenes, respectively, had similarity values of more than 60 % (Additional file 1: Figure S1). The top hit species for both of the sand lizard datasets were similar. More than half of the top hits from each transcriptome dataset were for genes from *Anolis carolinensis*, and many of the rest were from turtles and birds (Fig. 1b). This is consistent with a closed phylogenetic relationship among lizards, birds and turtles [23].

**Table 2:** Functional annotation of unigenes

		*P. erythrurus*		*P. putjatia*	
	**Database**	Number	Percent(%)	Number	Percent(%)
**Annotated**	NR	29,454	51.81	32,556	51.56
	Swiss-Prot	27,458	48.3	30,575	48.42
	COG	8,562	15.06	9,505	15.05
	KEGG	2,593	4.56	2,762	4.37
	GO	20,993	36.93	23,294	36.89
	Total	29,495	51.89	32,823	51.98
**Unannotated**		27,350	48.11	30,317	48.02
**Total**		56,845	100	63,140	100

The GO category distributions of the unigenes for both species were highly similar (Fig. 1c). The three main functional categories in each case were ‘Biological process’ (represented by 3,799 unigenes from *P. erythrurus* and 4,235 from *P. putjatia*), ‘Cellular component’ (4,037 from *P. erythrurus* and 4,552 from *P. putjatia*), and ‘Molecular function’ (3,854 from *P. erythrurus* and 4,218 from *P. putjatia*). Large groups of unigenes from both species (13,697 and 15,219 from *P. erythrurus* and *P. putjatia*, respectively) were assigned to all three categories. The COG database was used to define the orthologous functions of the identified unigenes. The distributions of unigene function categories for the two sand lizards were also very similar (Fig. 1d).

### Phylogenetic analysis

Lizard phylogenetic trees presented in previous publications were generally constructed on the basis of mitochondrial DNA sequence data and/or a few nuclear genes [24–26]. The development of transcriptome sequencing technologies in recent years has made it possible to determine phylogenies more efficiently than using traditional PCR- and EST-based methods [27]. Several publications have demonstrated the utility of transcriptome data for resolving the relationships of *Spalacidae* [28], *Passerida* [29], and the large tetrapod group consisting of turtles, birds and crocodiles [23].

**Fig. 2:**
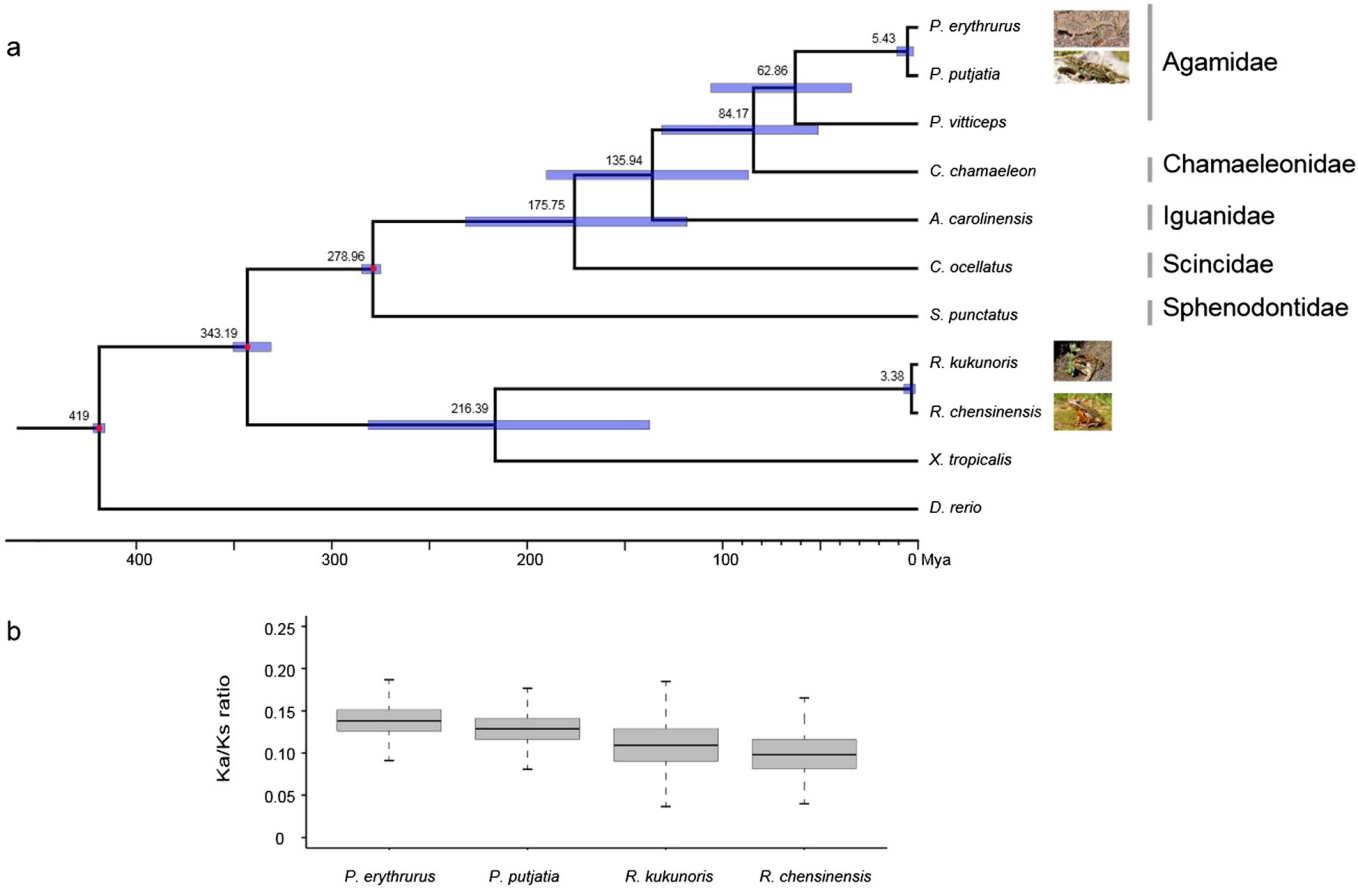
Divergence time and the ratio of Ka/Ks for the researched species. **(a)** Divergence time estimates for the major lizard clades generated using MCMCtree and the 4-fold degenerate sites. The red dots correspond to calibration points. Divergence time estimates (Mya) are indicated above the appropriate nodes; blue nodal bars indicate 95 % confidence intervals. **(b)** The box plot shows the ratio of non-synonymous to synonymous mutations (Ka/Ks) for *P. erythrurus, P. putjatia, R. kukunoris* and *R. chensinensis* branches

Given the limitations of previously reported phylogenetic trees for major lizard families, we sought to construct a more reliable tree to support subsequent analyses. A phylogenomic approach was adopted based on a dataset consisting of our sequence information for the toad-headed lizards *P. erythrurus* and *P. putjatia* together with data for five other lizards, three amphibians, and the zebra fish as an outgroup. A phylogenetic tree was constructed on the basis of four-fold degenerate sites among 209 single copy genes shared across these 11 species (Additional file 2: Figure S2). The bootstrap support for all nodes was 100 % and the identified phylogenetic relationships were consistent with those reported previously [24, 25]. Divergence times were estimated for each node, indicating that *P. erythrurus* and *P. putjatia* are closely related and diverged around ~5.43 Mya (million years ago, Fig. 2a and Additional file 3: Table S1). This estimated divergence time is similar to that reported previously (3 ~ 6 Mya) [16] and the divergence time of each node in the new tree was comparable to that reported at the TimeTree website (http://www.timetree.org) (Additional file 3: Table S1). This result represents the first attempt to characterize the phylogenetic relationships and divergence times of lizards on the basis of transcriptomic data generated by RNA-Seq. Our results provide a reliable phylogenetic tree for lizards and demonstrate the power of RNA-Seq as a tool for studying phylogeny.

### Hypotheses concerning mechanisms of high altitude adaptation in *P. erythrurus*

**Fig. 3:**
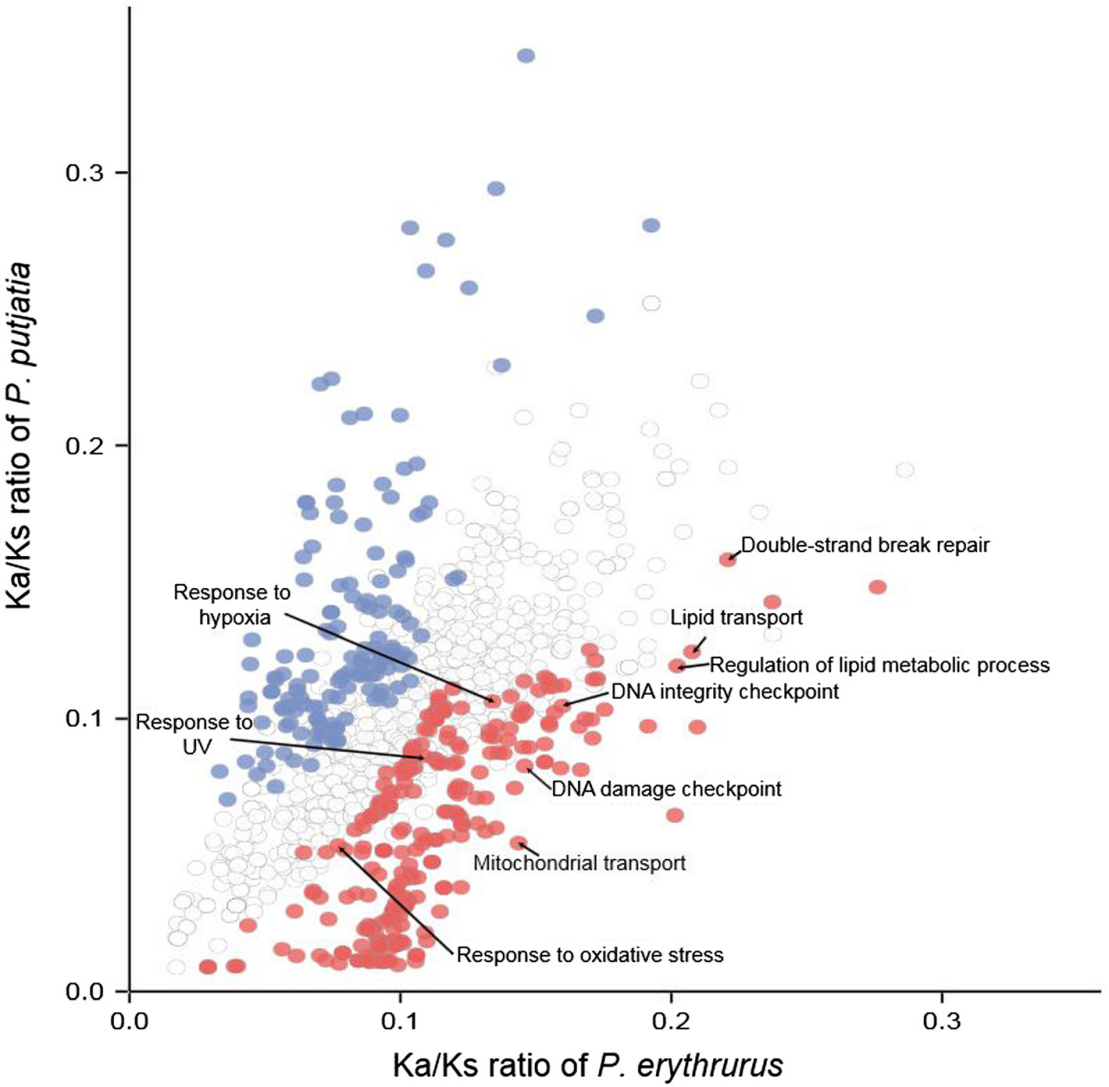
Comparison of Ka/Ks ratios for *P. erythrurus* and *P. putjatia* with respect to different GO functional categories. Blue dots represent categories with an elevated evolutionary rate along the *P. putjatia* lineage. Red dots represent categories with an elevated evolutionary rate along the *P. erythrurus* lineage. A full list of the categories is presented in Additional file 4: Table S2

Adaptive divergence at the molecular level may be reflected by an increased rate of non-synonymous changes within genes involved in adaptation [7]. We used our phylogenetic tree in conjunction with a branch model constructed in the PAML software package to determine the Ka, Ks, and Ka/Ks values for 4,094 separate alignments of *P. erythrurus* and *P. putjatia*. The results showed that the mean value of Ka/Ks ratio in *P. erythrurus* was higher than that in *P. putjatia* (P < 2.2 × 10^−22^, binomial test, Fig. 2b). A total of 206 GO categories showing accelerated evolutionary rate (P < 0.05, binomial test) were detected in *P. erythrurus* and 128 in *P. putjatia* (Additional file 4: Table S2 and Fig. 3). In *P. erythrurus*, the accelerated categories were mainly linked to three functional groups potentially associated with high altitude adaptation. The first functional group was related to hypoxia tolerance, consisting of the GO categories “response to hypoxia” and “response to oxidative stress”. The second functional group was energy metabolism, which was associated with GO categories including “mitochondrial transport”, “ATP metabolic process”, “lipid transport”, “regulation of lipid metabolic process”, and “steroid metabolic process”. The third functional group was response to UV damage, and included the GO categories “response to UV”, “DNA damage checkpoint”, “DNA integrity checkpoint”, and “double-strand break repair”.

By combining our results with those of earlier studies, we were able to tentatively infer the mechanisms that underpin adaptation to high altitude in *P. erythrurus*. The partial pressure of oxygen decreases with increasing altitude, so organisms living at high altitude must be tolerant of chronic hypoxia [1]. This tolerance is established in two ways, the first of which is based on increasing the capacity to take up and transport oxygen [30]. Genes associated with the “response to hypoxia” promote oxygen binding and transport to facilitate the capture of oxygen from the air. In particular, the HIF-1α pathway contributes to hypoxia tolerance by activating the expression of vascular endothelial growth factor to promote the formation of new blood and thereby maintain an adequate supply of oxygen [31]. The capillary density and blood hemoglobin concentration of the high-altitude lizards are both significantly greater than those of their low-altitude counterparts, further increasing their capacity for oxygen transport [32]. The second way of adapting to the low-oxygen conditions associated with high altitudes is to reduce oxygen consumption. This typically involves reducing the rates of metabolic processes and ATP demand (hypometabolism) [30]. Metabolic suppression entails reducing the mitochondrial rate while maintaining a core temperature high enough to permit survival and supplying enough ATP to sustain core physiological functions. This necessitates an increase in mitochondrial efficiency such that more ATP and heat are generated while consuming less oxygen. ATP production in such cases is largely driven by aerobic respiration, and lipid oxidation accounts for a relatively high proportion of the organism’s energy supply [20]. The finding that genes associated with energy metabolism and lipid metabolic process show accelerated divergence in *P. erythrurus* may thus reflect adaptation to high altitude. In addition to needing an increased capacity for oxygen uptake and transport, and a reduced metabolic rate with increased mitochondrial efficiency, high altitude dwelling organisms must cope with higher levels of UV radiation than their lowland counterparts. UV exposure can damage DNA molecules by generating highly reactive chemical intermediates such as oxygen radicals [33]. This presents a particularly acute challenge to reptiles, which often rely on basking for thermoregulation [34]. This increased level of DNA damage appears to have caused accelerated divergence of genes associated with DNA damage checking and DNA repair in *P. erythrurus*. While these findings are intriguing, it should be noted that analyses based on GO enrichment and computational approaches for detecting genes with accelerated evolutionary rates can yield high false-positive rates. Consequently, additional functional and physiological experiments are needed to verify the contributions of the identified genes to high altitude adaptation [35].

### Detecting candidate genes under positive selection in *P. erythrurus*

To identify genes that are likely to be important in the high altitude adaptation of *P. erythrurus,* we used rigorous criteria to identify candidate positively selected genes (PSGs) in the lineage. Previous studies that attempted to detect genes subject to positive selection in specific lineages often had worryingly high false positive rates due to the use of overly loose criteria [36–38], so rigorous criteria were employed in this work to obtain more robust results. We initially used the PRANK (Probabilistic Alignment Kit) to align the orthologs, which is considered to be more accurate than alternative methods and to provide a lower level of false positives [39]. Rather than performing simple pairwise comparisons of Ka/Ks ratios for *P. erythrurus* and *P. putjatia*, we used the branch-site likelihood ratio test to generate a list of candidate PSGs [40]. This approach was shown to have reasonable power and an acceptable false positive rate under a variety of selection schemes. We then manually removed all candidate PSGs with potential errors in their alignments to minimize the false discovery rate. After applying these strict criteria, 43 candidate high altitude responsive genes remained on the list for *P. erythrurus* (Additional file 5: Table S3).

**Fig. 4:**
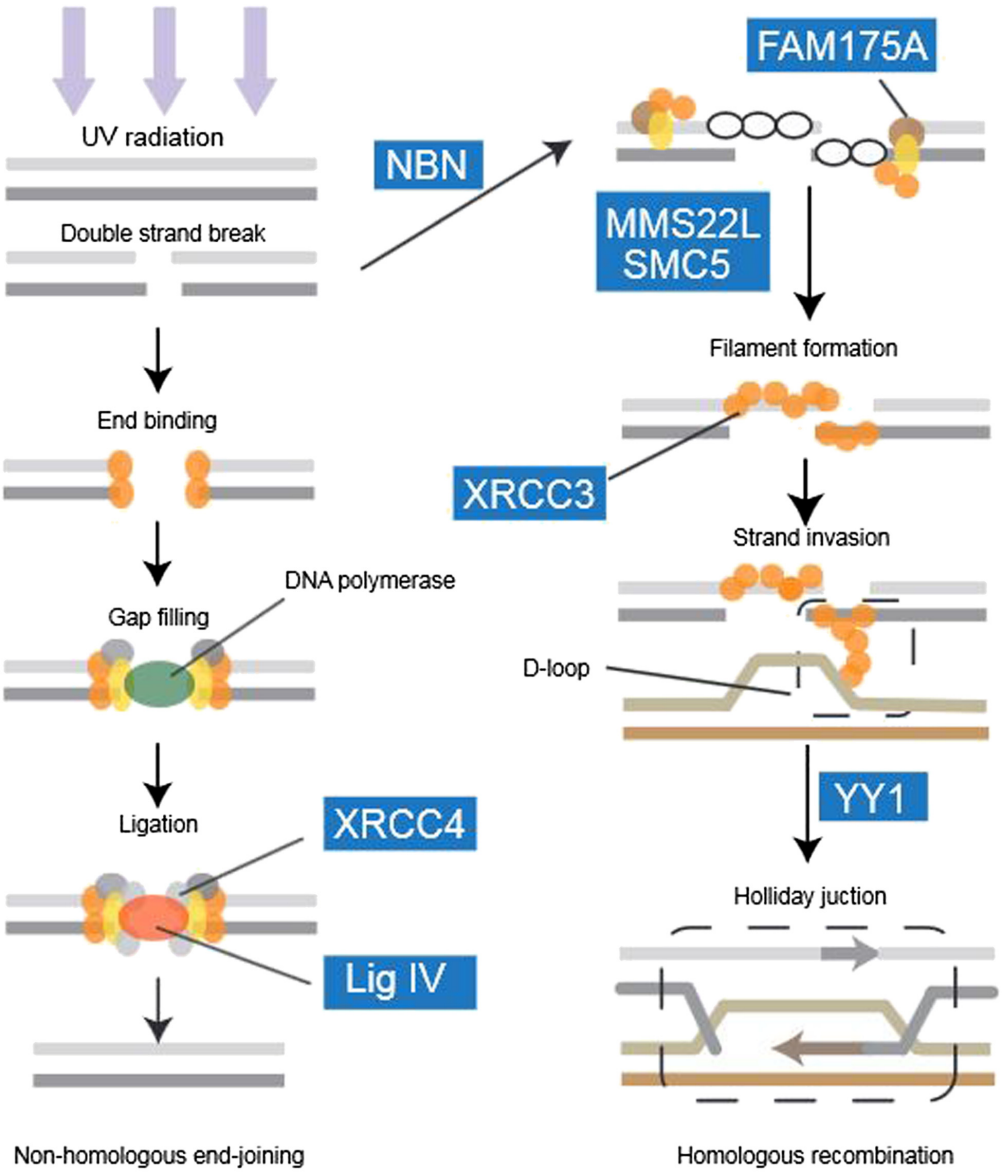
The DNA double-strand break repair pathway. Candidate PSGs along the *P. erythrurus* lineage are highlighted in blue

The functions of these candidate PSGs were consistent with the three functional groups of GO categories exhibiting accelerated evolution. Two of the candidate PSGs, *AGTRAP* and *UBE2D1*, are both involved in the response to hypoxia, corresponding to the first functional group of GO categories. *AGTRAP* encodes proteins that interact with the angiotensin II type I receptor to negatively regulate angiotensin II signaling [41], which may partially attenuate hypoxia-induced pulmonary hypertension [42]. *UBE2D1* enables the ubiquitination of the hypoxia-inducible transcription factor HIF-1α by interacting with the E1 ubiquitin-activating enzyme and the E3 ubiquitin-protein ligases [43, 44]. Another two candidate PSGs, *AOPB* and *NDUFB6***,** can be linked to the second group of GO categories, which relate to energy metabolism. *NDUFB6* encodes a component of the mitochondrial membrane respiratory chain [45] while *AOPB* plays a key role in lipid transport [46]. Finally, ten of the candidate PSGs are linked to the repair of DNA damage and the third group of GO categories. Two of these candidate genes (*RFC1* and *DSCC1*) encode components of the replication factor C complex that loads PCNA onto DNA during the S phase of the cell cycle, and are thus associated with mismatch repair [47, 48]. The other 8 candidate PSGs in this group have roles in the repair of DNA double-strand breaks (DSBs) (Fig. 4). The protein products of *Lig 4* and *XRCC4* combine to form the Lig4-XRCC4 complex, which ligates the ends of the two DNA strands during the final step of DSB repair by non-homologous end-joining [49, 50]. The *NBN* and *FAM175A* encode proteins that transduces the DNA damage signal and functions as an effector, both of them could verify the presence of DSBs and recruiting DNA repair proteins to the site of DNA damage during the early stages of homologous recombination repair [51–53]. The *SMC5* and *MMS22L* genes encode proteins that promote the recruitment of XRCC3 and other RAD51 paralogs to form the RAD51 filament**,** which appears to be a key event driving strand invasion [54–56]. *YY1* encodes a protein that plays a key role in binding the intermediate structures (Holliday junctions) during DNA synthesis [57].

**Fig. 5:**
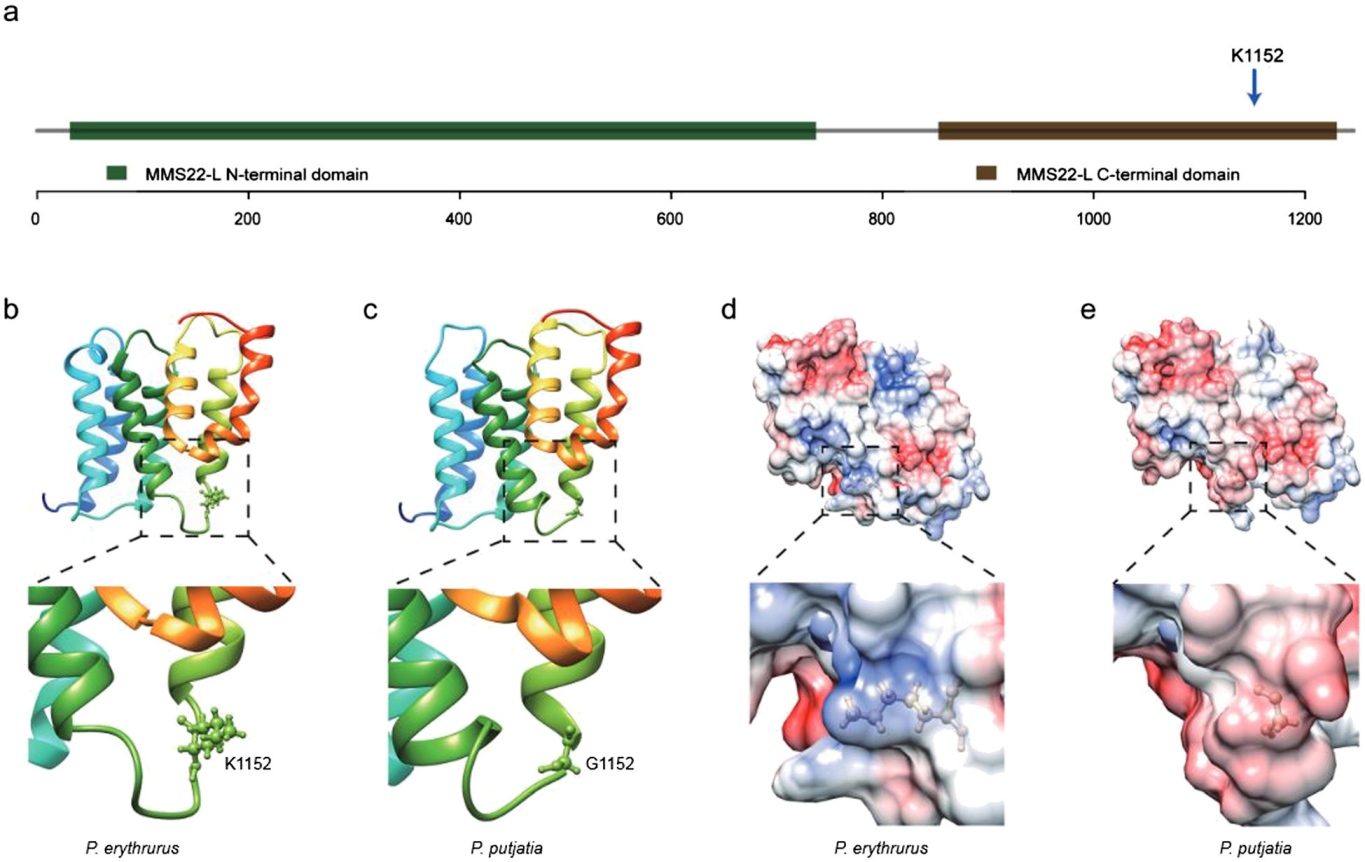
The structure of MMS22L protein sequences.**(a)** The MMS22L protein domain. The green frame represents the large N-terminal domain of MMS22L, while the brown frame shows its C-terminal portion. The candidate positively selected site is indicated by blue arrow. **(b, c)** Ribbon representation of Pseudo-atomic models of the MMS22L C-terminal domain in the *P. erythrurus* and *P. putjatia*, respectively. From the N-terminus to the C-terminus, it was colored from blue to red in a rainbow way. The zoomed-in picture showed the ball and stick representation of the mutated residues, which located at the loop region. **(d, e)** Electrostatic potential map of MMS22L C-terminal domain in the *P. erythrurus* and *P. putjatia*, respectively. The range of electrostatic surface potential is shown from −7 kT/e (red color) to +7 kT/e (blue color). The local electrostatic potential around the mutated residues was zoomed-in at the bottom panal.

We also investigated the amino acid sequence mutations observed in these 14 candidate PSGs from *P. erythrurus* lineages, their likely impact on the three-dimensional structures of the corresponding proteins, and the potential relationships between the structural changes and functional properties. Unfortunately, there was only one candidate PSG (*MMS22L*) for which can be aligned to a suitable template in the PDB data bank to build a homology model from the Phyre2 server. The candidate positively selected amino acid mutation (G1152K) in this modeled sequence is located within a conserved C-terminal domain (PF14911) (Fig. 5a). We generated a pseudo-atomic homology model of the MMS22L protein, which features seven α-helices connected by six short loops to form a compact functional domain (Fig. 5b,c). When we zoomed in the fourth connecting loop which connects the fourth and fifth α-helices, the residue G1152 is located quite adjcent to the N-termial of the fifth α-helix. Since the glycine mutated to a positively charged lysine, it indeed changed the regional electrostatic potential from slightly negative one to the almost entire positive evironment as the comparison of the respective electrostatic potential maps show to us (Fig. 5d,e). We speculate that the G1152K substitution altering its affinity for TONSL and the activity of the resulting complex in the recombination-dependent repair of collapsed replication forks [56]. In addition, there were 29 other candidate PSGs whose functions could not be immediately linked to high altitude adaptation on the basis of literature data. Further studies will be required to clarify the roles of these genes in high altitude tolerance.

### Convergent evolution in ectothermic animals adapted to life at high altitudes

Convergence is the independent evolution of similar features in different species. Its occurrence supports the hypothesis that specific environmental challenges can induce species to evolve in predictable and repeatable ways [58]. Studies on convergence between native animals living at high altitudes was very important to understanding the mechanisms of adaptation to high altitude. Genes associated with three distinct functions (response to hypoxia, energy metabolism and response to UV damage) were found to have been subject to adaptive selection in *P. erythrurus* during its adaptation to life at high altitude. We therefore sought to determine whether adaptation based on these functions is common in poikilothermic organisms living at high altitudes.

One notable early study on high altitude adaptation involved a pair of ranid frog species, the high altitude dwelling *Rana kukunoris* and the low altitude dwelling *R. chensinensis* [22]. We reanalyzed the sequence data for these two species to identify GO categories exhibiting accelerated divergence using the protocol developed for use with the toad-headed lizards. *R. kukunoris* was found to have a higher branch Ka/Ks ratios than *R. chensinensis* (P < 2.2 × 10^−22^, binomial test, Fig. 2b). 255 GO categories showing putatively accelerated evolutionary rates (P < 0.05, binomial test, Additional file 4: Table S2) were detected in *R. kukunoris*, with functional annotations similar to those identified in *P. erythrurus*. Thus, GO categories associated with the functions response to hypoxia, energy metabolism, and response to UV damage were all found to exhibit accelerated divergence in the high altitude dwelling *R. chensinensis.* Specifically, the GO categories “response to hypoxia” and “response to oxidative stress” were associated with the function of response to hypoxia; the categories “respiratory electron transport chain”, “regulation of lipid metabolic process”, “glycerophospholipid metabolic process” and “lipid biosynthetic process” were associated with energy metabolism; and the categories “response to UV”, “DNA integrity checkpoint”, “DNA damage checkpoint”, and “single-stranded DNA binding” were associated with the function of response to UV damage. We also detected 49 candidate PSGs along the *R. kukunoris* lineage (Additional file 5: Table S3), two of which (*UBE2D1* and *NBN***)** overlap with candidate PSGs for the *P. erythrurus* lineage. The overlap presumably reflects convergent evolution relating to the response to hypoxia and DNA damage repair. This finding is consistent with previous studies that have demonstrated convergent evolution associated with hypoxia adaptation in different high-altitude Andean hummingbirds [59] and in Tibetan Mastiffs and Tibetan people [11].

Studies on two separate pairs of high- and low-altitude poikilothermic species from the QTP revealed significantly higer Ka/Ks ratios for high altitude species than for their low altitude counterparts, which suggests that the high altitude species may have experienced adaptive evolution that allows them to cope with their extremely inhospitable environment. However, the elevated Ka/Ks ratios could also be due to a relaxation of selective pressure; further population genomic analyses will be required to test this alternative hypothesis. We identified three main function groups showing evidence of accelerated evolution, suggesting that genes from these categories may play key roles in adaptation to high altitude life among poikilotherms. While we were writing this article, another paper on high altitude adaptation in lizards was published in which the highland dwelling *P. vlangalii* was compared to its lowland relative *P. przewalskii* [60]*.* That work similarly indicated that genes associated with responses to hypoxia and UV damage had undergone adaptive evolution in the high altitude species, which is consistent with our findings and suggests that the mechanisms of adaptation identified for *P. erythrurus* may be common to many highland-dwelling cold-blooded organisms. To properly test this hypothesis, it will be necessary to perform additional functional and physiological experiments that should be integrated with genomic/transcriptomic analyses of a wider range of high altitude-dwelling poikilothermic species [13, 14].

## Conclusion

We have successfully sequenced and annotated large-scale, multi-tissue, multi-individual transcriptomes for two ectothermic vertebrate species and enriched the expressed sequence data available for the genus *Phrynocephalus*. The transcriptome data sets reported here represent a valuable resource that will support further expressional and functional studies that will help interested researchers to address ecological and evolutionary questions concerning sand lizards and other lizard species. High altitude adaptation is a complex process that involves numerous genes. We identified several functional groups and genes that may have undergone accelerated evolution and positive selection in the highland-dwelling *P. erythrurus* and *R. kukunoris* lineages. Similar GO categories with accelerated evolutionary rates and candidate positively selected genes were identified in both species. In addition, three main gene functions that might contribute to high altitude adaptation were identified: responses to hypoxia, energy metabolism, and responses to UV damage. We hypothesise that these three functions are generally important in adaptation to high altitude life among poikilotherms. The findings presented herein increase our understanding of the mechanisms by which cold blooded animals adapt to highland life and will support further studies on high altitude adaptation among lizards, other reptiles, and poikilothermic species in general.

## Methods

### Ethics statement

All animal specimens were collected legally. Animal collection and utility protocols were approved by the Ethics Committee of Animal Experiments at Lanzhou University and in accordance with guidelines from the China Council on Animal Care.

### Sample collection

Adult *P. putjatia* individuals were captured in June, 2013, from Gui’de County at an altitude of 2,312 m above sea level (m.a.s.l). Adult *P. erythrurus* individuals were captured in July, 2013 from the Tuotuo River at an altitude of 3,557 m.a.s.l. To include as many expressed genes as possible, we sampled seven different tissues from one male and one female of each species, including brain, heart, liver, kidney, testes/ootheca, lung and skeletal muscle. Tissue samples for RNA extraction were stored at −70 °C in a cryogenic refrigerator following euthanasia and dissection.

### Sequencing

Total RNA was isolated using the TRIzol reagent (Invitrogen, Carlsbad, CA, USA) and the RNeasy Kit (Qiagen, Hilden, Germany). A single pooled RNA sample was prepared for each species by mixing equal volumes of the RNA extracted from each tissue sample. These pooled samples were then used for cDNA preparation and RNA-Seq. cDNA library construction and sequencing were performed by Encode Genomics (Suzhou, China). RNAs with poly(A) tails were purified from total RNA using oligonucleotide (dT) magnetic beads and fragmented into short sequences that were used to template cDNA synthesis by PCR. The resulting cDNA libraries were purified using a PCR extraction kit (QiaQuick) and had an insert size of 200–700 base pairs (bp). The paired ends of the library were sequenced with a read length of 90 bp using the Illumina HiSeq 2000 sequencing platform. The image data generated by the sequencer were converted into raw sequence data by base calling to yield the raw reads, which were stored in the FastQ format. The entire process followed a standardized procedure and was monitored using a standard Quality Control System. All reads were deposited in the NCBI Short Read Archive (SRA) under accession number SRP050887.

### Data filtration and *De novo* assembly

We first cleaned the raw sequence reads by removing the exact duplicates from both sequencing directions with the FASTX Toolkit [61]. We further trimmed reads by removing adapter sequences, reads for which unknown base calls (N) represented more than 5 % of all bases, low complexity sequences, and low quality sequences (i.e. sequences for which >45 % of the bases were called with a quality score of ≤7). *De novo* assembly of the clean reads was performed using the Trinity software package, which efficiently constructs and analyses sets of *de Bruijn* graphs and then fully reconstructs a large fraction of transcripts, including alternatively spliced isoforms and transcripts from recently duplicated genes [62]. The calculations were performed using the –CuffFly option, which enforces the use of the cufflinks-like algorithm to report minimum transcripts. The transcripts were then clustered by CD-HIT-EST [63], with a sequence similarity cutoff of 95 %. The RSEM package [64] within Trinity was used to obtain abundance estimates for the transcriptome assemblies. The final assemblies were produced after deleting transcripts with FPKM values below 1 to ensure that all of the included transcripts could be detected. Partial and complete open reading frames (ORFs) were predicted using the TransDecoder script present in the Trinity package, with a minimum length of 100 amino acids.

### Functional annotation

Unigenes and predicted protein sequences were used as queries to search protein databases using the BLAST program [65]. Queries were performed against the NCBI (National Center for Biotechnology Information) NR (non-redundant database), Swiss-Prot, COG (cluster of orthologous groups databases) and KEGG (Kyoto Encyclopedia of Genes and Genomes) databases. Homology searches were conducted using BLASTx with an e-value cut-off of 1E-05 (in the case of the COG database, 1E-20 was used for increased stringency). Gene ontology (GO) terms were obtained from NR hits using the Blast2GO program [66] with default parameters for the mapping and annotation steps, except that an e-value cutoff of 1E-6 was applied and only the first 20 hits from the BLAST process were considered. GO functional classifications were obtained for all unigenes using the WEGO (Web Gene Ontology Annotation Plot) software [67]. COG function classification analysis of all unigenes sequences was performed using in-house Perl scripts. The metabolic pathways were mapped using the KAAS (KEGG Automatic Annotation Server) [68] with a bi-directional best-hit strategy to assign KEGG orthology terms (KO) to unigenes. The identified pathways were settled using their respective KO assignments.

### Ortholog identification and sequence alignment

To explore the phylogenetic relationships of the major lizards, characterize the mechanisms of adaptation to high altitude in *P. erythrurus*, and determine whether there was any evidence of functional convergent evolution among highland-dwelling poikilotherms, we downloaded sequence data for five lizard species, three amphibians, and the zebra fish as an outgroup. For the three genome-sequenced species (*Anolis carolinensis, Xenopus tropicalis* and *Danio rerio*), the CDS and protein sequences from the Ensemble database were downloaded (release 74). For the four transcriptome-sequenced lizards (*Pogona vitticeps, Chamaeleo chamaeleon, Chalcides ocellatus,* and *Sphenodon punctatus*), the unigenes were downloaded from the supplementary data for the corresponding publications. For the two transcriptome-sequenced ranid frogs (*Rana chensinensis* and *Rana kukunoris*)*,* sequencing reads were downloaded and processed using Trinity to assemble the transcripts, after which Transdecode was used to extract CDS and protein sequences. Additional file 6: Table S4 provides details of websites from which all of these datasets can be downloaded.

Orthologs were identified using InParanoid [69] and MultiParanoid [70] by considering protein sequences from 11 species (*A. carolinensis, X. tropicalis, D. rerio, P. vitticeps, C. chamaeleon, C. ocellatus, S. punctatus, R. chensinensis*, *R. kukunoris, P. erythrurus* and *P. putjatia*). In-house Perl scripts were used to identify high-confidence 1:1 orthologs based on rigorous criteria: genes in each cluster with confidence scores of less than 1 were removed and only clusters containing a single gene from each species were selected. All orthologs were aligned using PRANK [71] with the codon option, and alignments shorter than 150 bp after removing sites with ambiguous data were discarded. Finally, we obtained two multiple sequence alignments: (1) one alignment (9,175 bp, 10.1 % of total length of the 206 genes ) for the concatenated 4D-sites of the 206 high-confidence single-copy genes among 11 species, which was used to support the phylogenetic analysis and estimate divergence times; and (2) 4,094 individual alignments for each high-confidence single-copy gene within six of the studied species (*Xenopus tropicalis, Danio rerio, P. erythrurus, P. putjatia, Rana chensinensis* and *Rana kukunoris*) and at least one of the other five species (*Anolis carolinensis, Pogona vitticeps, Chamaeleo chamaeleon, Chalcides ocellatus* and *Sphenodon punctatus*), which was used to investigate the mechanisms of adaptation to high altitude.

### Phylogenetic analysis

The jModelTest program [72] was used to select the best fitting substitution model according to the Akaike information criterion based on the 9,175 bp concatenated 4D-sites. The GTR + I + G model was determined to be the best fitting, and the Phyml program [73] was employed to build the ML tree. The bootstrap support for all nodes was 100 %. Based on this phylogenetic tree, we estimated the divergence time of each node by the MCMCtree program in the PAML package [74] and the BEAST program [75]. For the MCMCtree program, we set the clock parameter to 1 to ensure the use of a strict molecular clock. Two-round analyses were performed in which the first 5,000 iterations were discarded as a burn-in followed by sampling every 50 iterations until 20,000 samples had been gathered. These two-round analyses consistently yielded convergence; one representative set of obtained results is presented in this work. For the BEAST program, a strict clock was assumed and the analyses were run for 10,000,000 generations, sampling 1 generation in every 1,000; the first 1,000,000 generations were discarded as a burn-in. For both programs, the divergence times were calibrated (on the assumption of a constant molecular clock) against the timing of the divergences of the Zebra fish from the lineage leading to toads/lizards (421.75- 416 Mya), the toads from the lineage leading to lizards (350.1-330.4 Mya), and Sphenodon punctatus from the lineage leading to other lizards (275–285 Mya). The timings of the first two divergences were obtained from http://www.fossilrecord.net/ and that of the third from http://www.timetree.org/. The results obtained using the two program were similar; see Additional file 3: Table S1 for more details.

### Analysis of accelerated evolutionary rates along each lineage

We used the Ka/Ks ratios to measure the evolutionary rate along a lineage. The values of Ka and Ks and the Ka/Ks ratios were estimated for each of the 4,094 single-copy orthologs using the Codeml program with the free-ratio model for each branch [76]. 10000 concatenated alignments constructed from 150 randomly chosen orthologs were used to estimate lineage-specific mean values. Based on the GO classifications, the unique orthologs were clustered into different functional terms and the Ka, Ks and Ka/Ks ratios for each term was calculated. A comparison of evolutionary rates based on non-synonymous substitution between *P. erythrurus* and *P. putjatia* or *R. chensinensis* and *R. kukunoris* was conducted using a binomial test (see [77] for details of the method used). Only GO categories containing more than 20 genes were included in this analyses.

### Detecting candidate genes under positive selection

To clarify the mechanism of adaptation to high elevations, we used the Codeml program from the PAML package with a branch-site model [40] to detect positively selected genes in any of the *P. erythrurus*, *P. putjatia*, *R. chensinensis* or *R. kukunoris* lineages, with each lineage being specified as the foreground branch sequentially. Very rigorous criteria were used to identify the PSGs. In the branch-site model, a PSG was required to have a Ka/Ks ratio greater than 1 in the model with positive selection on the foreground branch and to have positive sites for the foreground branch with a posterior probability in excess of 0.95 based on the Bayes empirical Bayes (BEB) results [78]. A likelihood ratio test was also conducted to compare the model with positive selection on the foreground branch to a null model with no positive selection on the foreground branch for each orthologous gene, and only those genes with *P* values of less than 0.05 were selected [38, 40]. We then manually removed all PSGs with potential errors in their alignments to minimize the false discovery rate.

### Protein structure homology modeling

We investigated the relationships between the amino acid sequence mutations and the structure modeling within 14 candidate PSGs, which were associated with response to hypoxia, response to UV and energy metabolism. These candidate PSGs’ functional domains were obtained from the Pfam database [79] and the protein structures were constructed with the Phyre2 server, which uses the alignment of hidden Markov models via HHsearch to significantly improve the accuracy of alignment and detection rates [80]. We downloaded all of the high confidence results from the Phyre2 server and constructed ribbon representation of the structures with UCSF Chimera version 1.10.1 [81]. The electrostatic potentials at the surfaces of the predicted protein structures were rendered using PyMol [82] with ABPS and PDB2PQR [83].

### Availability of supporting data

The transcriptome reads of *P. erythrurus* and *P. putjatia* have been deposited in the NCBI Short Read Archive (SRA) database under the accession number SRP050887, while the links that can be used to download information concerning the other 9 species considered in this work were list in the Additional file 6: Table S4. The data of the phylogenetic analysis are available in the Dryad Digital Repository: http://dx.doi.org/10.5061/dryad.f587b [84].

## Additional files

Additional file 1: Figure S1. Characteristics of the gene annotations for the assembled transcripts obtained by searching the NR database. **(a)** E-value distribution of the BLASTx hits for each transcript, based on an E-value cut-off of 1E-5. **(b)** Similarity distribution of BLASTx hits for each transcript.

Additional file 2: Figure S2. Maximum likelihood tree for 11 species. Phyml with the GTR + I + G model and 4-fold degenerate sites was used to construct the phylogenetic tree. Bootstrap support for all nodes was 100 %. (PDF 100 kb)

Additional file 3: Table S1. Estimated divergence times for specific nodes in this work and previous studies.

Additional file 4: Table S2. GO categories showing accelerated evolutionary rates within the *P. erythrurus* and *R. kukunoris* lineages.

Additional file 5: Table S3. Candidate PSGs from the *P. erythrurus* and *R. kukunoris* lineages.

Additional file 6: Table S4. Links that can be used to download information concerning the 9 species considered in this work.
