# Supplementary material for: Comparative transcriptomic analysis revealed adaptation mechanism of Phrynocephalus erythrurus, the highest altitude Lizard living in the Qinghai-Tibet Plateau
Source: BMC Evol Biol. 2015 Jun 2;15:101. doi: 10.1186/s12862-015-0371-8 (PMC4450828; doi:10.1186/s12862-015-0371-8)
Supplement: Supplementary file 1 — Characteristics of the gene annotations for the assembled transcripts obtained by searching the NR database. (a) E-value distribution of the BLASTx hits for each transcript, based on an E-value cut-off of 1E-5. (b) Similarity distribution of BLASTx hits for each transcript. [file 12862_2015_371_MOESM1_ESM.pdf]

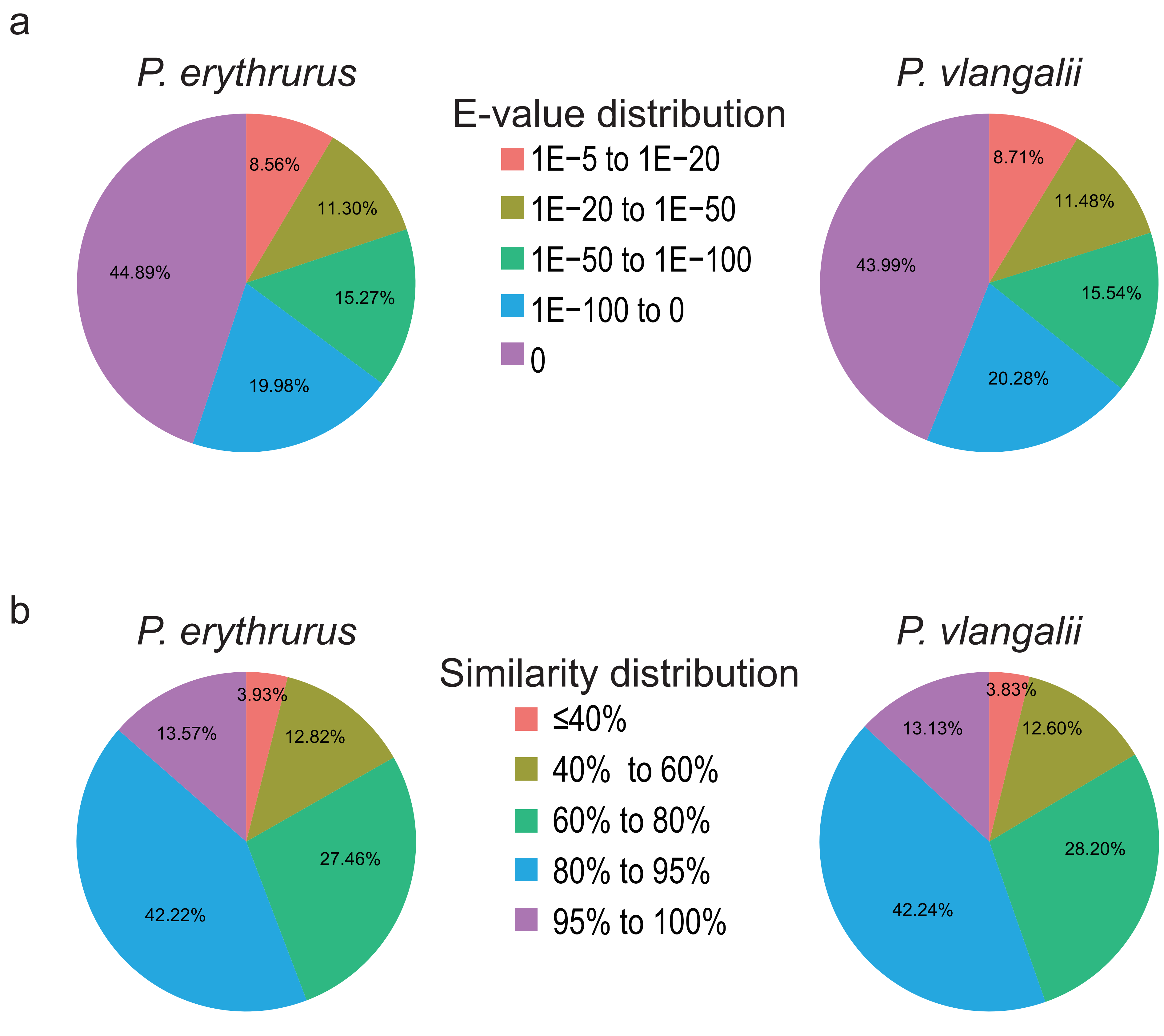
a
P. erythrurus
E-value distribution
1E−5 to 1E−20
1E−20 to 1E−50
1E−50 to 1E−100
1E−100 to 0
0
8.56%
11.30%
15.27%
19.98%
44.89%
P. vlangalii
8.71%
11.48%
15.54%
20.28%
43.99%
b
P. erythrurus
Similarity distribution
≤40%
40% to 60%
60% to 80%
80% to 95%
95% to 100%
3.93%
12.82%
27.46%
42.22%
13.57%
P. vlangalii
3.83%
12.60%
28.20%
42.24%
13.13%
