# Supplementary figures and images for: Comparative transcriptomic analysis revealed adaptation mechanism of Phrynocephalus erythrurus, the highest altitude Lizard living in the Qinghai-Tibet Plateau
Source: BMC Evol Biol. 2015 Jun 2;15:101. doi: 10.1186/s12862-015-0371-8 (PMC4450828; doi:10.1186/s12862-015-0371-8)

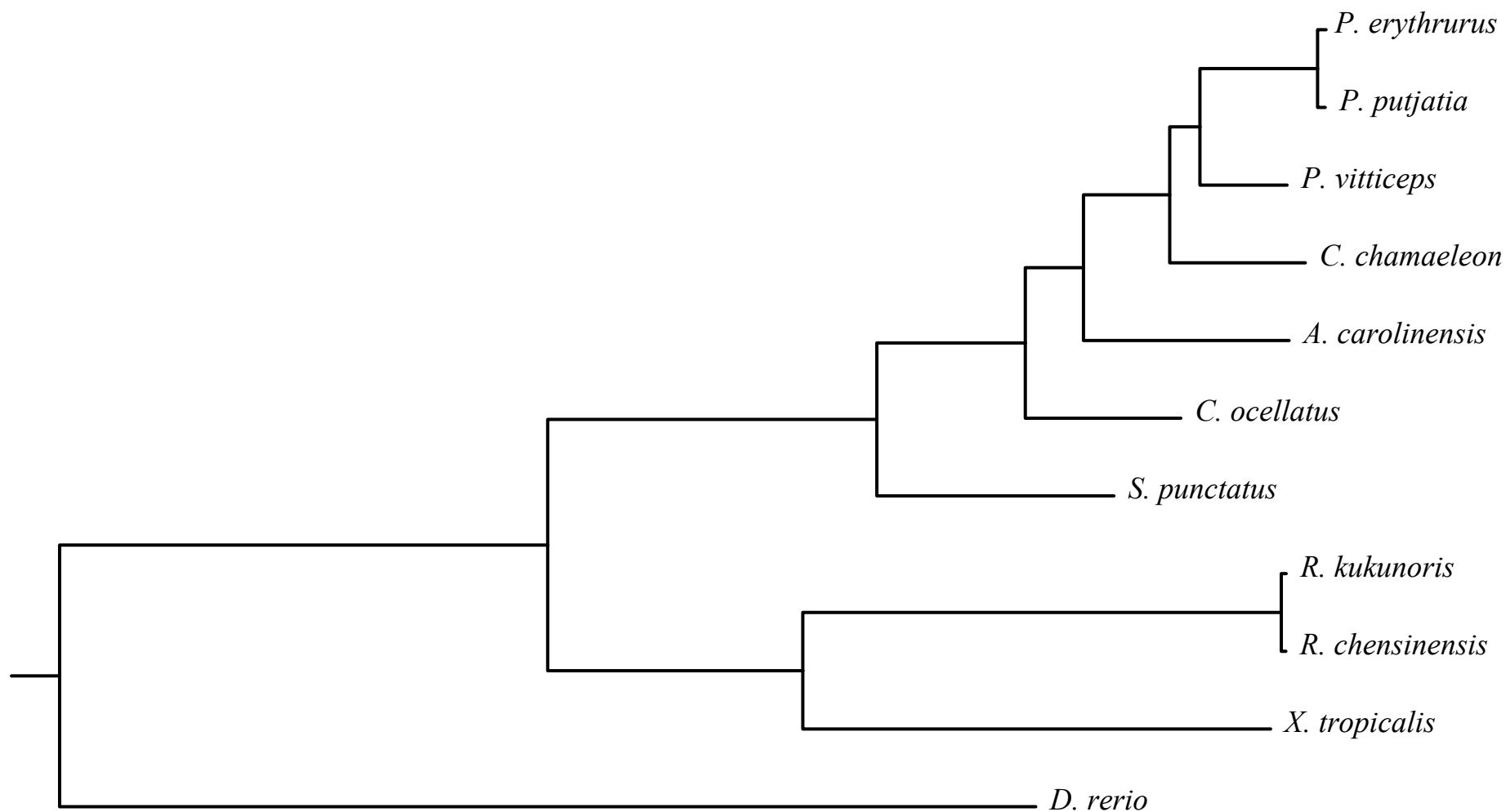

0.3 substitutions / site

Supplement: Supplementary file 2 — Maximum likelihood tree for 11 species. Phyml with the GTR + I + G model and 4-fold degenerate sites was used to construct the phylogenetic tree. Bootstrap support for all nodes was 100 %. (PDF 100 kb) [file 12862_2015_371_MOESM2_ESM.pdf]
